# Successful CRISPR/Cas9 mediated homologous recombination in a chicken cell line

**DOI:** 10.12688/f1000research.13457.2

**Published:** 2018-05-30

**Authors:** Ekaterina Antonova, Olga Glazova, Anna Gaponova, Aykaz Eremyan, Svetlana Zvereva, Natalya Grebenkina, Natalya Volkova, Pavel Volchkov

**Affiliations:** 1 Moscow Institute of Physics and Technology, Dolgoprudny, Moscow Region, 141701, Russian Federation; 2 Ernst Institute of Animal Husbandry, Podolsk Municipal District, Moscow Region, 142132 , Russian Federation

**Keywords:** CRISPR/Cas9, targeting, homology directed repair, chicken DF-1 cell line, endogenous promoter tandem expression

## Abstract

**Background:** CRISPR/Cas9 system is becoming the dominant genome editing tool in a variety of organisms. CRISPR/Cas9 mediated knock out has been demonstrated both in chicken cell lines and in chicken germ cells that served to generate genetically modified birds. However, there is limited data about CRISPR/Cas9 dependent homology directed repair (HDR) for avian, even in cell culture. Few attempts have been made with integrations in safe harbor loci of chicken genome that induces constitutive expression of the inserted gene. Gene expression under an endogenous promoter would be more valuable than under a constitutive exogenous promoter, as it allows the gene expression to be tissue-specific.

**Methods:** Three gRNAs were chosen to target chicken 3’-untranslated region of GAPDH gene. Cas9-mediated activity in the targeted locus for the gRNAs in DF-1 cells was estimated by T7E1 assay. To edit the locus, the HDR cassette was added along with CRISPR/Cas9. The inserted sequence contained eGFP in frame with a GAPDH coding sequence via P2A and Neomycin resistance gene ( *neoR*) under cytomegalovirus promoter. Correct integration of the cassette was confirmed with fluorescent microscopy, PCR analysis and sequencing. Enrichment of modified cells was done by G418 selection. Efficiency of integration was assessed with fluorescence activated cell sorting (FACS).

**Results:** We have established a CRISPR/Cas9 system to target an endogenous locus and precisely insert a gene under endogenous control. In our system, we used positive and negative selection to enrich modified cells and remove cells with undesirable insertions. The efficiency of CRISPR/Cas9-mediated HDR was increased up to 90% via G418 enrichment. We have successfully inserted eGFP under control of the chicken GAPDH promoter.

**Conclusions:** The approach can be used further to insert genes of interest under control of tissue-specific promoters in primordial germ cells in order to produce genetically modified birds with useful for biotechnological purposes features.

## Introduction

Genetically modified chickens have great potential in agriculture, industry, biological research and pharmaceuticals [ Farzaneh *et al*., 2017; Lyall *et al*., 2011; Nishijima & Iijima, 2013; Schock *et al*., 2016]. Precise and effective genome editing is one of the most important aspects in creating genetically modified organisms. Traditionally, transgenic chickens were generated using retroviruses [ von Werder *et al*., 2012]. However, retroviral delivery of an inserted sequence is an ineffective method due to random distribution of integration sites, and has adverse effects. Clustered regularly interspaced short palindromic repeat (CRISPR), and CRISPR-associated nuclease (Cas9) - CRISPR/Cas9 are now widely used as an efficient method for genetic modification in a wide variety of organisms [ Bassett *et al*., 2013; Cong *et al*., 2013; Friedland *et al*., 2013; Hu *et al*., 2013; Hwang *et al*., 2013; Li *et al*., 2013; Liang *et al*., 2015; Mali *et al*., 2013; Nakayama *et al*., 2013; Niu *et al.*, 2014; Wang *et al*., 2013; Wagner *et al*., 2014]. Cas9 cuts double stranded DNA at the site specified by the guide RNA (gRNA). The double strand break can be repaired in an error-prone way by non-homologous end joining (NHEJ), leading to small insertions/deletions, or by homology-directed repair (HDR), when a donor DNA-template is added [ Hsu *et al*., 2014; Merkert & Martin, 2016]. Nuclease-mediated gene insertion is several orders of magnitude more efficient compared with spontaneous recombination of DNA template alone [ Lin *et al*., 2014; Zhang *et al*., 2017] that makes CRISPR/Cas9 an effective tool for genome editing.

Although CRISPR/Cas9-mediated gene editing has been widely used in a lot of organisms, this tool still has been rarely applied in avian species. There are some examples of successfully generated genetically modified chickens with usage of TALEN nuclease (Transcription Activator-Like Effector Nuclease) [ Park *et al*., 2014; Taylor *et al*., 2017]. Meanwhile CRISPR/Cas9 was used only to knock out genes in poultry ( *in vivo* experiments) [ Oishi *et al*., 2016]. The CRISPR/Cas9 tool is a novel instrument compared with TALEN and some aspects of successful targeting with the Cas9 nuclease still need to be elucidated for avian species. However, the ability not only to knock-out genes, but also to induce a tissue-specific expression of a gene of interest without destroying its endogenous locus would be beneficial.

Here, we show the system to precisely insert a gene under the control of an endogenous promoter and select the cells with the successful integration. The result indicates that this system can be used as a tool for chicken genome editing.

## Methods

### Construction of expression vectors

We used human codon-optimized Cas9 (hCas9) as it has been previously demonstrated that the optimized Cas9 works in chicken cells and there was no need to synthesize chicken codon-optimized nuclease [ Bai *et al*., 2016; Véron *et al*., 2015; Wang *et al*., 2017; Zuo *et al*., 2016]. A plasmid CAG-Cas9 (#89995, Addgene; Cambridge MA, USA) was taken for human codon-optimized Cas9 expression. Similarly as for Cas9 it has been previously demonstrated that the human U6 promoter works in chicken cells [Bai Y; Lee *et al.*, 2017; Véron *et al*., 2015]. Unique 20bp sequences for the selected gRNAs were cloned under human U6 promoter in the plasmid phU6-gRNA (#53188, Addgene). A plasmid pQE30TaqRFP (Evrogen; Moscow, Russia) coding RFP was used for cotransfection as a reporter of the efficiency of DNA delivery to the cells.

### Targeting vector design

**Figure 1.**
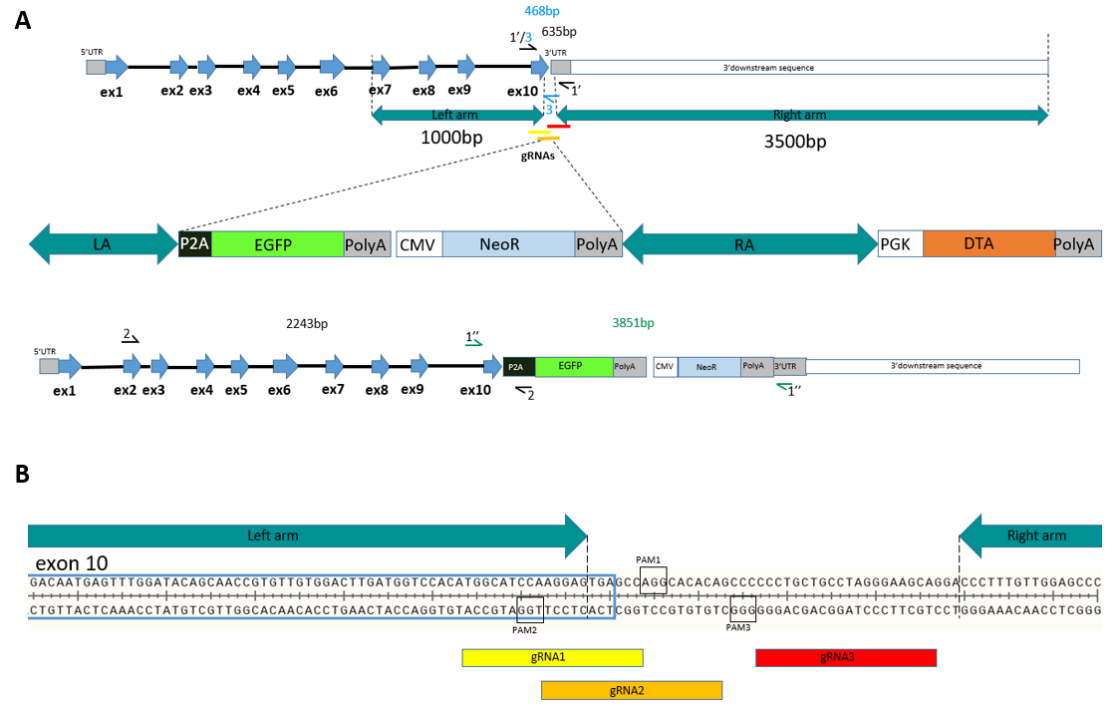
( **A**) A schematic illustration of the chicken GAPDH locus, HDR-cassette, edited GAPDH locus. ( **B**) A schematic diagram of the target sites in the chicken GAPDH 3’UTR flanked with homology arms.

The targeting vector was designed based on the plasmid LSL-Cas9-Rosa26TV (#61408, Addgene). Homology regions of 999bp and 3093bp for left and right arms respectively were amplified from the genomic DNA of chicken cell line DF1 by PCR, and cloned using MauBI and PmeI restriction sites for the left arm, and SgrDI and AscI for the right arm respectively (DF-1 genome is yet to be sequenced, common chicken genomic data is available here). Left and right homology regions in the shuttle flank the P2A-eGFP sequence, where eGFP is enhanced green fluorescent protein. Coding sequence of the left arm was in frame with P2A-eGFP in order to provide the gene transcription under the control of endogenous promoter. Neomycin resistance (neo) gene was cloned after eGFP under a constitutive cytomegalovirus (CMV) promoter for positive selection of cells with the desired insertion. The total length of the inserted sequence between two homology arms in the shuttle was 3259bp. Diphteria Toxin Fragment A (DTA) coding sequence was inserted in the shuttle after the right arm under a PGK promoter for negative selection ( Figure 1A). The left homology arm was amplified by PCR with the following primers: 5’-TTGTTGACCTGACCTGCCGTCTGGAG-3’ and 5’-CTCCTTGGATGCCATGTGGACCATCAAG-3’; the right homology arm was amplified with primers 5’-CCCTTTGTTGGAGCCCCTGCTCTTC-3’ and 5’-GAGCCCTGTATCTTCCTTGCACAGACC-3’. The primer were designed in Primer-BLAST and synthesized by Evrogen.

Length of the right homology arm is longer in order to increase HDR due to high repeat content in 3’URT region. Chicken right and left homology arms are separated by a stretch of 42bp in the beginning of 3’UTR that is targeted by the gRNAs ( Figure 1B). Thus, CRISPR/Cas9 cutting of successfully edited genomic DNA is prevented due to the targeted sequence not being present in the vector for HDR.

### gRNA selection

We searched for gRNAs to target the GAPDH locus in the 3’UTR within 50bp near the stop codon of the gene. We sequenced exon 10 of GAPDH and the beginning of the 3’UTR. 100bp region of the chicken GAPDH around the stop-codon was analyzed on the ChopChop server for gRNA design. We selected three gRNAs in the locus ( Table 1, Figure 1B).

**Table 1.** Selected gRNAs. gRNAs target sequences and PAMs are shown. gRNA - guide RNA; PAM - Protospacer adjacent motif.

gRNA	gRNA sequence	PAM
gRNA	TGGCATCCAAGGAGTGAGCC	AGG
gRNA	TGTGTGCCTGGCTCACTCCT	TGG
gRNA	TGCTTCCCTAGGCAGCAGGG	GGG

These gRNAs had a few predicted off-target sites ( Table S1). None of the off-target sites were present in a known coding sequence of the Gallus gallus genome.

### DF1 cell line cultivation

The chicken DF-1 cell line (ATCC, CRL-12203) was cultivated in DMEM medium (Gibco, ThermoFisher) containing 10% fetal bovine serum (FBS, Gibco) and 100u/ml penicillin/streptomycin mix according to ATCC recommendation. Cells were maintained at 37°C with 5% CO2. For experiments, DF-1 cells were plated into 6-well or 24-well-plates.

### Cell transfection

Cells were co-transfected with 4µg DNA plasmid mix for 6-well plates or 1µg for 24-well plates using TurboFect transfection reagent (ThermoFisher; Waltham MA, USA). The plasmid mix contained the Cas9 plasmid, the gRNA plasmid, the RFP plasmid and the linear HDR shuttle. We used the equimolar ratio of all components: pCas9:pgRNA:pRFP:HDR shuttle. After 72 hours post transfection, cells were analyzed by fluorescence microscopy. Five – seven technical repeats were made for every experiment, which were used for genome analysis, G418 selection and for flow cytometry analysis.

### Selection with geneticin

Titration of geneticin (G418, Gibco) on the DF-1 cell line had been performed before selection and the optimal concentration 500ng/µl was chosen. Cells were cultured with geneticin for 10 days with daily medium change. Geneticin-resistant cells were analyzed with PCR to confirm the correct insert. Fluorescence microscopy and FACS were used to visualize and define the percentage of eGFP-positive cells respectively.

### Cell viability and cytotoxicity assays

For evaluation of an appropriate concentration of geneticin, CellTiter-Blue® Cell Viability Assay (Promega, Fitchburg WI, USA) was used. The principle of the assay is based on conversion of resazurin to resorufin by metabolically active cells that results in the generation of a fluorescent shift from 605 nm to 573 nm. Thus, the produced fluorescence is proportional to the number of viable cells. 48-well assay plate containing cultured cells with media was set up. Three technical repeats were performed for each concentration at each time point. G418 (Gibco) in concentrations of 100ng/µl; 200ng/µl; 500ng/µl; 1000ng/µl; 2000ng/µl and 5000ng/µl was added. The recommended volume of CellTiter Blue Reagent was added to a series of wells at 48, 96, 140, 192 and 240 hours following geneticin treatment. After addition of the reagent, cells were incubated for 2 hours. Fluorescence was measured (CLARIOstar - BMG Labtech; Offenburg, Germany) at 560/590nm. 570–600nm absorbance versus concentration of G418 was plotted.

### Detection of Cas9-mediated cuts in the targeted locus

For detection of Cas9-mediated cuts, a T7E1 assay was performed [ Kim *et al.*, 2009]. The assay is based on the ability of the T7 Endonuclease to recognize and cleave non-perfectly matched DNA. DF-1 cells were harvested and genomic DNA was isolated using the Wizard® Genomic DNA Purification Kit (Promega). PCR products were amplified using the primers T7-for: 5’-GACCATTTCGTCAAGCTTGTTTCC-3’ and T7-rev: 5’-GATCAGTTTCTATCAGCCTCTCCCAC-3’. The primer were designed in Primer-BLAST and synthesized by Evrogen. The amplified product, 635bp in length, was purified from agarose gel. 200ng of the product was reannealed to form heteroduplex DNA at the following temperature conditions: 95°C – 5min; 95°C-85°C with ramping 2°C/sec; 85°C-25°C with ramping 0,1°C/sec; 25°C – 2min; 4°C. - After the reannealing, T7 nuclease EI (New England Biolabs; Ipswich MA, USA) was added (10 units). Heteroduplex DNA was incubated with the enzyme at 37°C during 30 min. The resulting product was analyzed by electrophoresis.

Mutation frequencies were calculated as described by Guschin *et al*. (2010) based on the band intensities. Band intensities were measured with ImageJ software (version 2). Mutation frequency (%) = 100 · [1 – (1 – F)1/2], where F represents the cleavage coefficient, which is the proportion of the total relative density of the cleavage bands to all of the relative densities of the cleavage bands and uncut bands [ Guschin *et al*., 2010].

### PCR analysis

To confirm HDR cassette integration several pairs of primers were used ( Figure S1D). The primers were designed based on schematic sequence of the edited locus in Figure S1D, checked in BLASTn for specificity and synthesised by Evrogen.

Primers forward: 5’-GACCATTTCGTCAAGCTTGTTTCC-3’ and reverse: 5’-GATCAGTTTCTATCAGCCTCTCCCAC-3’ amplify the area surrounding the site of insertion ( Figure S1D, primers pointed as 1’ and 1’’). PCR product in the case of insertion had a length of 3851bp. Amplified product from the endogenous locus without an integration had a length of 635bp.

Amplification with primers exon 2-forward: 5’ - AATGGGCACGCCATCACTATCTTC - 3’ and P2A-reverse: 5’ - TGGCCCGGGATTCTCTTCGA - 3’ results in product only in the case of successful HDR-mediated cassette integration ( Figure S1D, primers pointed as 2). Primers forward 5’-GACCATTTCGTCAAGCTTGTTTCC-3’ and reverse 5’-tggcccgggattctcttcgac-3’ ( Figure S4D, primers pointed as 3) were used for PCR analysis from the genome of cells enriched by drug selection after successful modification. The product is only amplified in case of an unmodified genome. This happens due to the reverse primer aligning to the 50bp area of 3’UTR that was targeted by gRNAs, but it does not align to the HDR vector itself.

### FACS analysis

FACS analysis (Accuri™ C6 Flow Cytometer, BD Biosciences; San Jose CA, USA) was used to estimate the proportion of eGFP- positive cells.

### Off-target analysis

Potential CRISPR/Cas9 off-target sites for selected guides are presented in Table S1. The off-targets were predicted by ChopChop tool. All off-target sites were located in introns or in intergenic loci.

### *In vitro* Cas9 cleavage

Primers for *in vitro* gRNA synthesis:

In order to make dsDNA template for RNA transcription the two following oligonucleotides were annealed.

-CRISPR R: (common primer for all targets, it contains the sequence for RNA scaffold synthesis) - the oligonucleotide was common for synthesis of all DNA templates coding a gRNA:

5’–AAAAGCACCGACTCGGTGCCACTTTTTCAAGTTGATAACGGACTAGCCTTATTTTAACTTGCTATTTCTAGCTCTAAAAC–3’-CRISPR F: (the oligonucleotide contains the T7 promoter and the specific target sequence). Both oligonucleotides have a 20nt complementary sequence that allows them to anneal.

CRISPR F GAPDH gRNA1 

5’–GAAATTAATACGACTCACTATA GGGGCATCCAAGGAGTGAGCC GTTTTAGAGCTAGAAATAGC–3’

CRISPR F GAPDH gRNA2 

5’–GAAATTAATACGACTCACTATA GGGTGTGCCTGGCTCACTCCT GTTTTAGAGCTAGAAATAGC–3’

CRISPR F GAPDH gRNA3 

5’–GAAATTAATACGACTCACTATA GGGCTTCCCTAGGCAGCAGGG GTTTTAGAGCTAGAAATAGC–3’

The oligonucleotide synthesis was ordered from Evrogen.

Full-length dsDNA template was made via PCR overlap of corresponding oligonucleotides

### *In vitro* transcription of gRNAs

HiScribe T7 High Yield RNA Synthesis Kit (E2040S, New England Biolabs) was used for *in vitro* RNA synthesis of gRNAs, as described in the manufacturer’s protocol. RNA was purified using the MEGAclear™ Transcription Clean-Up Kit (AM1908, ThermoFisher) following the protocol from the manufacturer.

### *In vitro* Cas9 digestion

*In vitro* digestion was made by Cas9 Streptococcus pyogenes (S. pyogenes; New England Biolabs) according to the protocol from the manufacturer. PCR product for analysis was amplified with following primers: forward 5’-GACCATTTCGTCAAGCTTGTTTCC-3’and reverse 5’-GATCAGTTTCTATCAGCCTCTCCCAC-3’.

***Droplet digital PCR (ddPCR).***

Probes description

We designed three kinds of probes. Probe 1 - a reference probe (VIC) located away from the editing site to count all genomic copies ( Figure S2I). GAPDH was used as a reference, having a single copy per genome [ Weiskirchen *et al*., 1993].

We designed three kinds of probes. Probe 1 - a reference probe (VIC) located away from the editing site to count all genomic copies ( Figure S2I). GAPDH was used as a reference, having a single copy per genome [ Weiskirchen *et al*., 1993].

Probe 2 - a (FAM) probe, located in the inserted sequence ( Figure S2I). Probe 3 - a (VIC) probe located in DTA gene of the cassette that does not insert into the locus or kill the cells in case of insertion ( Figure S2I). The nucleotide sequence of the probes and primers are shown in the Table 2.

**Table 2.** Probes and primer sets used in droplet digital PCR.

Primers sequence	Probe sequence	Fluorophore- quencher	Target
FOR: 5’-AATGGGCACGCCATCACTATCTTC-3’ REV: 5’-CCATTTGATGTTGCTGGGGTCAC-3’	5’-CTCCTCTTGCCACTCCAGAGGATGAAAGTA-3’	VIC-BHQ	GAPDH locus - reference
FOR: 5’-CCTCAGGTATGACAATGAGTTTGGA-3’ REV: 5’-CCTGCTTGTTTCAACAGGGAGA-3’	5’-GGTCCACATGGCATCCAAGGAGTTT-3’	FAM-BHQ	inserted sequence from HDR cassette

***Samples description.***

The following samples were used:

-DNA from DF1 cells transfected with linearised cassette only (negative control). The sample was taken in amount 20ng.

-DNA from geneticin enriched DF1 cells, isolated 1 month after HDR. The sample was taken in amount - 1ng and 20ng.

-Water was used as a no template control (NTC) to rule out cross-contamination.

We set up two repeats for each amount of DNA.

Cassette probe was used to determine the copy number of transgene in DF1 cells. GAPDH probe was used as the reference.

***ddPCR reaction preparation.***

The following reagents were mixed in 96 plate to make reaction:

ddPCR Supermix for Probes (#186-3026, Bio-Rad);

10 U of EcoRI (#R3101S, New England BioLabs);

Genomic DNA (DNA dilutions 1ng and 20ng were selected based on preliminary experiments);

The total volume of the reaction was 20µl.

Droplets were generated with 20μl of the premixed reaction and a QX200 Droplet Generator according to the manufacturer’s instructions (Bio-Rad) and transferred to a 96-well PCR plate for standard PCR on a CFX96 Touch™ Real-Time PCR Detector system (Bio-Rad).

The following cycling programs were used:

1) 95°C for 10 min;

2) 95°C for 30 s;

3) 59–63 °C (in depends on pair of primers and probe) for 1 min; repeat steps 2 and 3 for 40 times;

Optimal annealing temperature was determined empirically for each pair of primers and probe with a temperature gradient. After PCR amplification, each droplet provides an independent fluorescent positive or negative signal indicating the target DNA was present or not. The droplets were analysed with a QX200 Droplet Reader (Bio-Rad) with the selected option “absolute quantification”. Positive and negative droplets are counted for each samples, and the software calculates the concentration of target DNA as copies per microliter.

***Quantification of ddPCR data.***

QuantaSoft (version 1.7.4.0917) was used for quantification (Bio-Rad).

An appropriate threshold between the positive and negative droplets was applied manually based on the NTC wells. ddPCR software reads the positive and negative droplets in each sample and plots the fluorescence droplet by droplet. The fraction of positive droplets determines the concentration of the target in the sample. Software calculates the concentration of target DNA as copies per microliter. Then the copy number of an unknown target is calculated relative to a known reference. In our case we estimated the copy number of inserted sequence relative to GAPDH gene. The confidence intervals for each well are calculated by QuantaSoft based on Poisson distribution.

The formula used for Poisson modeling is:

Copies per droplet = –ln(1 – p)

where p = fraction of positive droplets.

The primers and probes were designed in Primer-BLAST. Primers were synthesised by Evrogen. Probes were ordered from Syntol (Moscow, Russia)

## Results

In the current research we have studied CRISPR/Cas9-mediated homology directed repair in an endogen locus for expression of an integrated gene under the control of the endogenous promoter.

The GAPDH locus was chosen as a commonly expressed constitutive gene. In order to make the expression of our inserted gene be controlled by the promoter, we decided to insert the gene just after the coding sequence of GAPDH ( Figure 1A). To target the 3’UTR of chicken GAPDH we selected and designed three gRNAs. Before starting experiments on a chicken cell line, all selected gRNAs were tested *in vitro* using recombinant Cas9 *S. pyogenes*. Cas9 in complex with one of the three gRNAs made cuts and produced lengths of cleaved products corresponded to the expected lengths ( Figure S3).

### Targeting 3’UTR of chicken GAPDH by Cas9

In order to test the effectiveness of targeting endogenous 3’UTR of GAPDH with the selected gRNAs in chicken cells, Cas9, gRNA plasmids, combined with the RFP plasmid, were co-transfected in DF-1 cells. Cas9 expression vector without a gRNA was used as the negative control. RFP expression was estimated at 72h after transfection ( Figure S4). Figure S2 is represented by seven technical repeats. The average level of transfection efficiency was more than 45%.

**Figure 2.**
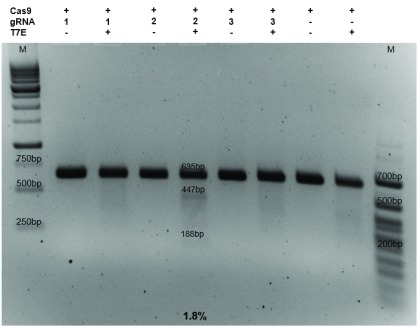
Detection of Cas9-mediated cuts in the targeted endogenous chicken GAPDH 3’UTR locus with T7 Endonuclease assay.

Genomic DNA was extracted from the cells, and T7 endonuclease I (T7EI) assay demonstrated that only gRNA2 in complex with Cas9 had activity in DF1 cells with the targeting rate around 1.8% ( Figure 2).

### Homologous recombination in GAPDH locus

**Figure 3.**
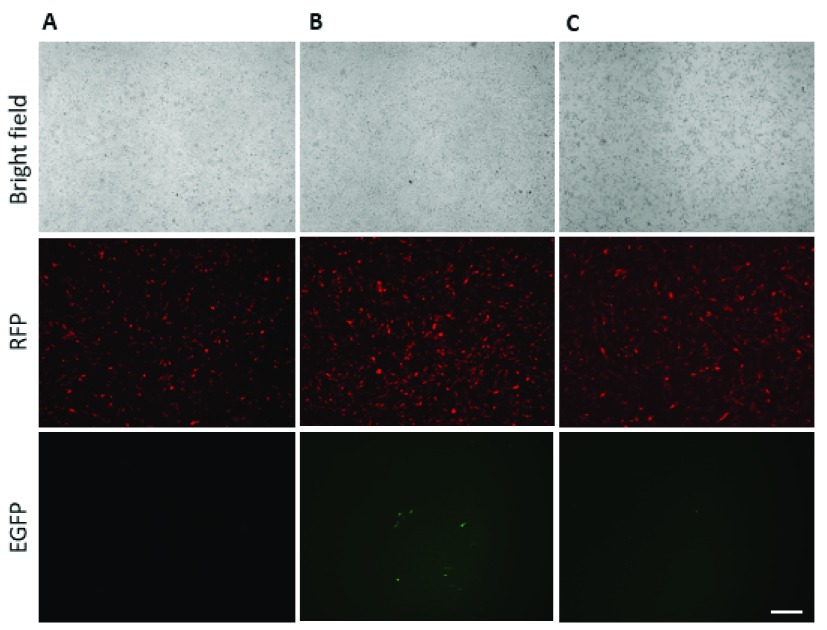
Homologous recombination at the CRISPR/Cas9-targeted 3’UTR GAPDH locus. ( **A**) Transfection with gRNA2, Cas9, RFP; ( **B**) Transfection with gRNA2, Cas9, RFP, cassette for HDR; ( **C**) Transfection with Cas9 without any gRNA, RFP, cassette for HDR. Scale bar = 200 µm.

In order to obtain cells with the insertion we co-transfected DF-1 cells with plasmids encoding Cas9, gRNA, RFP and cassette for homology directed repair. As a negative control we added Cas9, RFP and cassette without any gRNAs. At 72h after transfection we observed 0.5% GFP-positive cells of transfected cells in the experimental group ( Figure 3). Figure 3 is represented by five technical repeats. In the vector for HDR, eGFP does not have its own promoter and can be expressed only in the case of the correct insertion in frame with the GAPDH gene. The genomic DNA was extracted for PCR analysis. The analysis confirmed the presence of the expected integration ( Figure S1A and Figure S6B).

### Enrichment of successfully edited cells with geneticin selection

**Figure 4.**
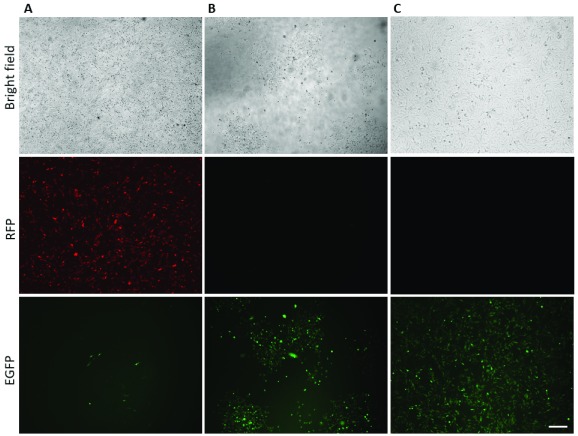
Enrichment of CRISPR/Cas9-modified cells with G418 selection, 500 ng/µl. ( **A**) 72h after transfection; ( **B**) 15 days after selection; ( **C**) 1 month after selection. Scale bar = 200 µm. Figure is representative of five technical repeats.

Applying drug selection in combination with CRISPR/Cas9 allowed us to select and grow colonies carrying the modification. Based on the survival curve ( Figure S5) we added 500 ng/µl of geneticin (G418) at 72h after transfection. The appropriate concentration of the drug was selected after titration in the DF-1 cell line ( Figure S5). Single eGFP-positive cells had developed in colonies after 10 days of incubation on G418. RFP fluorescence had vanished due to its transient expression ( Figure 4). Figure 4 is representative of five technical repeats.

One month after the drug selection the enriched cells were analyzed by PCR and FACS. PCR analysis confirmed the absence of cells without modification ( Figure S1C). FACS analysis showed about 90% of eGFP positive cells in the cell population ( Figure S6). The nucleotide composition around the insertion was analyzed by DNA sequencing, additionally confirming the correct integration ( Figure S7). Also we made a ddPCR analysis to measure the copy number of the integrated sequence per genome. DNA samples from enriched edited cells was analysed in comparison with DNA sample from the cassette only transfected cells (at 72 hour after transfection). 1-D plot with FAM positive droplets ( Figure S2.II.A), indicating insertion, and VIC positive ( Figure S2.II.B) droplets, indicating the reference gene GAPDH, plotted on the graph of fluorescence intensity versus droplet number is shown for each sample (please see the method description). The positive droplets determines the concentration of the target in the sample which are calculated into the concentration of target DNA as copies/µl. The number of copies of the insertion was normalised by the number of copies of GAPDH as it is known that birds have one copy of the gene [ Weiskirchen *et al*., 1993] that implies two alleles per genome. In result we got about 0,8 copy of the insertion per GAPDH allele ( Figure S2.III) that additional confirm the FACS data. The copy-number of DTA gene was also estimated to differentiate the cassette or improperly integrated sequence from the correct insertion. In the enriched edited cells DTA gene was not observed (data is not shown in the Figure S2.II). Thus, all used methodological approaches confirmed the correctness and effectiveness of the transgene integration.

Raw images of all gel images (Figure 2 and Supplementary Figure S1 and Supplementary Figure S3) Click here for additional data file. Copyright: © 2018 Antonova E et al. 2018 Data associated with the article are available under the terms of the Creative Commons Zero "No rights reserved" data waiver (CC0 1.0 Public domain dedication).

Plate reads for all time points performed for the cell viability assay (Figure S5) Click here for additional data file. Copyright: © 2018 Antonova E et al. 2018 Data associated with the article are available under the terms of the Creative Commons Zero "No rights reserved" data waiver (CC0 1.0 Public domain dedication).

FACs output files underlying Figure S7 Click here for additional data file. Copyright: © 2018 Antonova E et al. 2018 Data associated with the article are available under the terms of the Creative Commons Zero "No rights reserved" data waiver (CC0 1.0 Public domain dedication).

## Discussion

CRISPR/Cas9 is easy to use, specific, efficient, and multiplex [ Cong *et al*., 2013; Mali *et al*., 2013]. Here, we set up a system for efficient CRISPR/Cas9-mediated homologous recombination to successfully target chicken DF-1 cells. The approach can be used to obtain cell populations with a gene of interest under the control of a tissue-specific promoter. In this study, we targeted the 3’UTR area of chicken GAPDH. Successful attempts to target chicken genome with CRISPR/Cas9 have been reported. Mammalian codon-optimized Cas9 has been used to target PAX7 gene in chicken somatic cells [ Véron *et al*., 2015], PPAR-g, ATP5E, OVA genes [ Bai *et al*., 2016], myostatin gene [ Wang *et al*., 2017] in DF-1 cells, C1EIS gene [ Zuo *et al*., 2017] and Stra8 gene [ Zhang *et al*., 2017] in male germ cells. Mammalian codon-optimized Cas9 and chicken U6 promoter for gRNA were also used to target C2EIP gene in DF-1 and chicken embryonic cells [ Zhang *et al*., 2017; Zuo *et al*., 2016]. The researches demonstrated activity of mammalian adapted CRISPR/Cas9 in avian cells and the ability to knock out a gene. The CRISPR/Cas9 targeting efficiency in our experiments, according to T7E1 assay analysis, was around 1.8% that is similarly with the results from the previous article [ Bai *et al*., 2016]. The low effectivity in our case can be explained by several reasons: we were restricted by 50–100 bp sequence of genomic DNA for selection of gRNAs; the area of targeting is the beginning of 3’UTR, which usually has a lower GC content and has a lot of repeated elements; also the transfection efficiency of the DF1 cells with multiple plasmids was less than 50%. The effectiveness of targeting could be improved by increasing transfection effectiveness and applying surrogate reporter assay.

Traditionally homology directed recombination with long homology arms was used to insert a desired sequence at a desired place. For example, homology directed recombination at chicken JH segment was performed without CRISPR/Cas9 using ~8–9 kb of total homology arms. The inserted sequence had a size ~2000bp. The frequency of the insertion was low, about one targeted clone per 10 ^7^ transfected cells, and after drug selection it resulted in 28% of correctly targeted events [ Schusser *et al.,* 2013]. Thus, the approach has low effectiveness and accuracy.

It is known that applying Cas9 with a vector for homologous recombination enables the usage of 2kb of total homology instead of 7–8kb significantly increasing the effectiveness of an insertion. Several approaches to make homology directed repair in chicken cell line using CRISPR/Cas9 has been recently published. A stable genetic element in the chicken genome of the DF-1 cell, endogenous avian virus (EAV-HP), was targeted and the inserted sequence was 1200bp length [ Wang *et al*., 2017]. The EAV-HP is considered as a safe harbor, and can be used to generate constitutive expression of a gene of interest, although the element is contained in the chicken genome in multiple copies. The targeted effectiveness reached 49%. In the other article the Cas9 system was used for modifying the variable domain of the immunoglobulin heavy chain (IgH) in chicken PGCs *in vitro* [ Dimitrov *et al*., 2016]. The authors targeted a site approximately 300bp upstream of the translation initiation site of IgH using homology regions of 1133bp and 1011bp with the inserted sequence around 1500bp and had 33% of effectiveness after drug selection. Therefore, the researches demonstrated higher effectiveness of CRISPR/Cas9-mediated homology directed repair for constitutive expression of the inserted gene in chicken cells compared with traditional methods.

In our study we inserted into the GAPDH 3’UTR region a sequence up to 3000bp, that encoded the eGFP gene expressed under the control of an endogenous promoter and Neomycin resistant gene controlled by the CMV promoter. The sequence of 3’UTR and the following downstream sequence is not well characterized in chicken models [ International Chicken Genome Sequencing Consortium, 2004], that potentially could reduce the opportunity to design long homology regions required for a traditional gene targeting approach, so the CRISPR/Cas9 system is very helpful in the case. The length of homology regions has to correlate with the size of the insertion and we used homology regions 1000 and 3000bp. In practice amplification and cloning of the homology arms of such length was not labour-intensive and the same strategy of targeting a tissue-specific gene can be easily applied to other loci of interest. In our experiments HDR effectiveness at 72 hours after transfection was around 0.5% of RFP-positive cells in the case of targeting with gRNA2. Using drug selection, we achieved up to 90% targeted integration.

Expression from endogenous promoters could be favorable for other applications, for instance, synthesis of pharmaceutical proteins in the egg white under the ovalbumin promoter, or expression of a gene that provides a defense against a pathogen in tissues that are located in contact with the infection. Usage of an endogenous promoter to express an inserted gene also could prevent its epigenetic silencing. This is very important for long-term stable expression of pharmaceutical proteins and other applications. For now, genome modification in chickens has been established using germline stem cells, such as primordial germ cells (PGCs) [ Leighton *et al*., 2008; Song *et al*., 2014; van de Lavoir *et al*., 2006; van de Lavoir *et al.*, 2006]. We are planning to use our approach to insert a gene of interest, in place of eGFP, under the control of the tissue-specific promoter of PGC, and enrich cells after homology directed repair. Cultured PGCs can be transfected and injected into recipient-embryos, where they will produce the germline.

In conclusion, we demonstrated that the CRISPR/Cas9 system along with cassette for HDR can successfully target the 3’UTR of endogenous genes to integrate a gene under endogenous control.

## Data availability

The data referenced by this article are under copyright with the following copyright statement: Copyright: © 2018 Antonova E et al.

Data associated with the article are available under the terms of the Creative Commons Zero "No rights reserved" data waiver (CC0 1.0 Public domain dedication). 

Dataset 1: Raw images of all gel images ( Figure 2 and Supplementary Figure S1 and Figure S3) 10.5256/f1000research.13457.d192397 [ Antonova *et al*., 2018a]

Dataset 2: Plate reads for all time points performed for the cell viability assay ( Figure S4) 10.5256/f1000research.13457.d192398 [ Antonova *et al*., 2018b]

Dataset 3: FACs output files underlying Figure S5 10.5256/f1000research.13457.d192408 [ Antonova *et al*., 2018c]

Data underlying Figure S8 is available from Dataverse: http://dx.doi.org/10.7910/DVN/YSNKKC [ Antonova, 2018d]

Available under a CC0 - "Public Domain Dedication"

## Supplementary material

**Table S1. Predicted off-targets for each gRNA.**

**Figure S1. Confirmation of correct insertion with PCR.** ( **A**) The result of PCR analysis with primers 1’ and 1”; ( **B**) The result of PCR analysis with primers 2; ( **C**) The result of PCR analysis with primers 3; Scheme of GAPDH locus before and after HDR-mediated insertion with primers. 1. Genomic DNA from cells, transfected gRNA, Cas9; 2. Genomic DNA from cells, transfected gRNA, Cas9, HDR cassette; 3. Genomic DNA from cells, transfected Cas9 without gRNA, cassette; 4. H2O; 5. Cassette; 6. Genomic DNA from eGFP-positive 1 month since geneticin selection.

**Figure S2.** I. The scheme of genomic DNA before and after the cassette insertion with marked location of primers and probes for ddPCR. Probe 1 – VIC (green), Probe 2 - FAM(blue), Probe 3 - VIC(green). II. Representation of ddPCR results in 1-D plot for the following samples: Ex 20 ng DNA, Ex 1 ng DNA – DNA from DF1 cells isolated 1 month after HDR experiment following G418 selection; Control 20ng DNA – negative control, DNA from DF1 cells transfected with linearized cassette only; NTC- no template control. ( **A**) Amount of FAM positive droplets indicating the presence of the insertion. ( **B**) Amount of VIC positive droplets. VIC probes target GAPDH used as a reference to determine the copy number of insertion per genome. Positive droplets are those above the pink threshold line. III. The copy number of the insertion (transgene) per GAPDH allele. Experiment - DNA from DF1 cells cultivated during one month after HDR in presence of G418; Control – negative control, DNA from DF1 cells transfected with linearized cassette only.

**Figure S3. *In vitro* Cas9 digestion, using gRNA1, 2 and 3.** Cas9 with non-targeting scramble gRNA and Cas9 without any gRNA were used as negative controls.

**Figure S4. Evaluation of plasmid delivery efficiency in GAPDH targeting experiment.** ( **A**) Cas9, gRNA1, RFP transfection; ( **B**) Cas9, gRNA2, RFP transfection; ( **C**) Cas9, gRNA3, RFP transfection.

**Figure S5. Results of cell viability assays for G418 using the CellTiter-Blue Reagent.**

**Figure S6. Geneticin selection of eGFP-positive cells by flow cytometry analysis.**

**Figure S7. Sequencing of integrated HDR vector in the modified locus of GAPDH.** ( **A**) The coverage of sequenced area. ( **B**) Sequencing confirms in frame eGFP insertion.
