# Supplementary material for: Successful CRISPR/Cas9 mediated homologous recombination in a chicken cell line
Source: F1000Res. 2018 May 30;7:238. Originally published 2018 Feb 28. [Version 2] doi: 10.12688/f1000research.13457.2 (PMC6008848; doi:10.12688/f1000research.13457.2)
Supplement: Supplementary file 4 [file f1000research-7-16408-s0003.tgz › b4e1648e-c619-466f-b8c0-0de1894526ab.docx]

**SUPPLEMENTARY MATERIAL**

gRNA1

| Genomic location | Number of mismatches | Sequence (including mismatches) |
| --- | --- | --- |
| chr5:18491439 | 3 | CCTGGCTCgCTCCTgaGATGCCA |

gRNA2

| Genomic location | Number of mismatches | Sequence (including mismatches) |
| --- | --- | --- |
| chr14:3263303 | 2 | CCAAGGAGaGAGCCAGGCACAgA |

| Genomic location | Number of mismatches | Sequence (including mismatches) |
| --- | --- | --- |
| chr15:6211502 | 3 | TGCTTgCCTAGcCAGCAGtGGGG |
| chr26:4378652 | 3 | CCCagCTGCTGCCTtGGGAAGCA |

**Table S1. Predicted off-targets for each gRNA.**
