# Supplementary figures and images for: Successful CRISPR/Cas9 mediated homologous recombination in a chicken cell line
Source: F1000Res. 2018 May 30;7:238. Originally published 2018 Feb 28. [Version 2] doi: 10.12688/f1000research.13457.2 (PMC6008848; doi:10.12688/f1000research.13457.2)

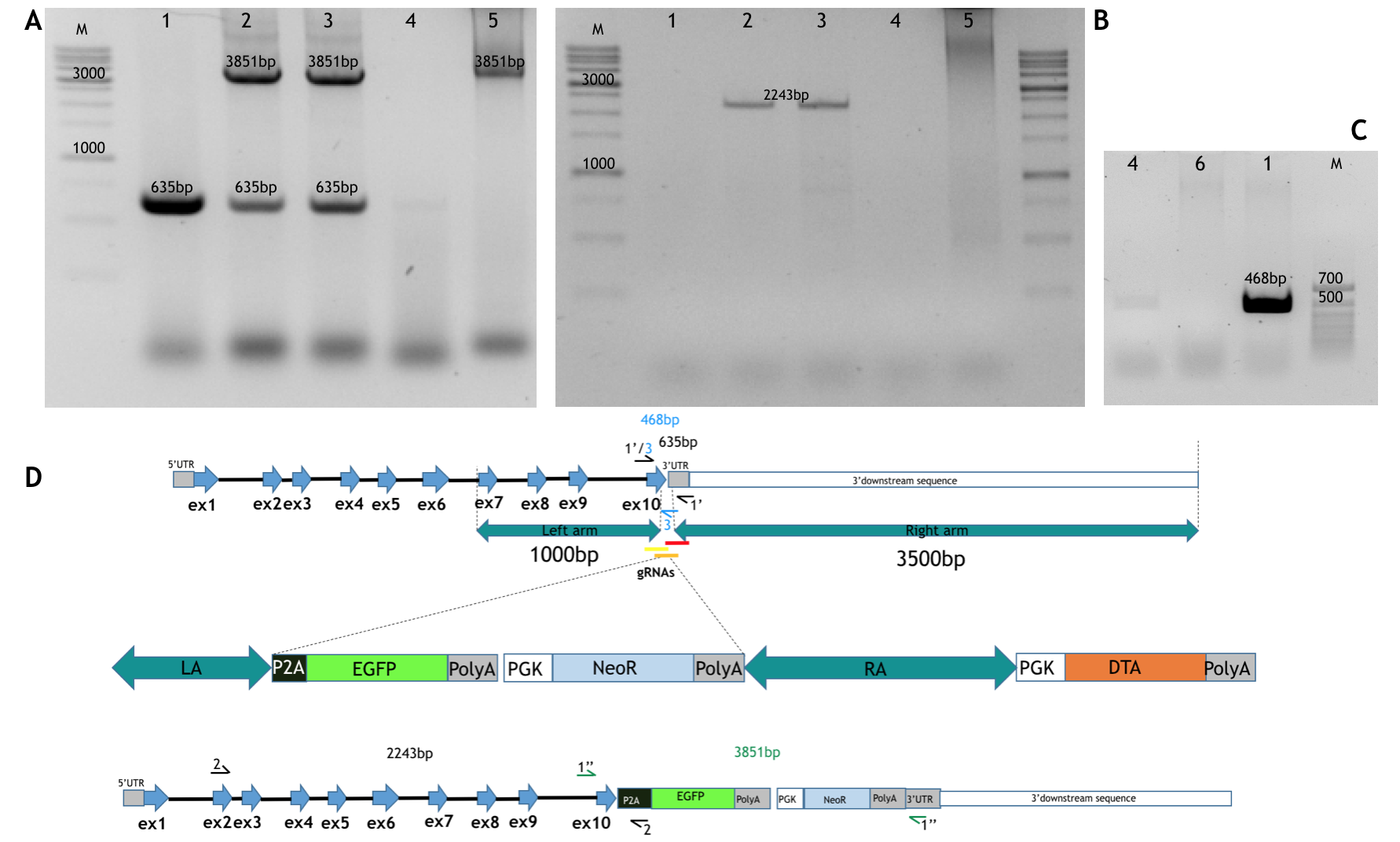

Supplement: Supplementary file 5 [file f1000research-7-16408-s0004.tgz › b39e3f52-8cd9-4e66-aae4-b8ec8384bad8.png]

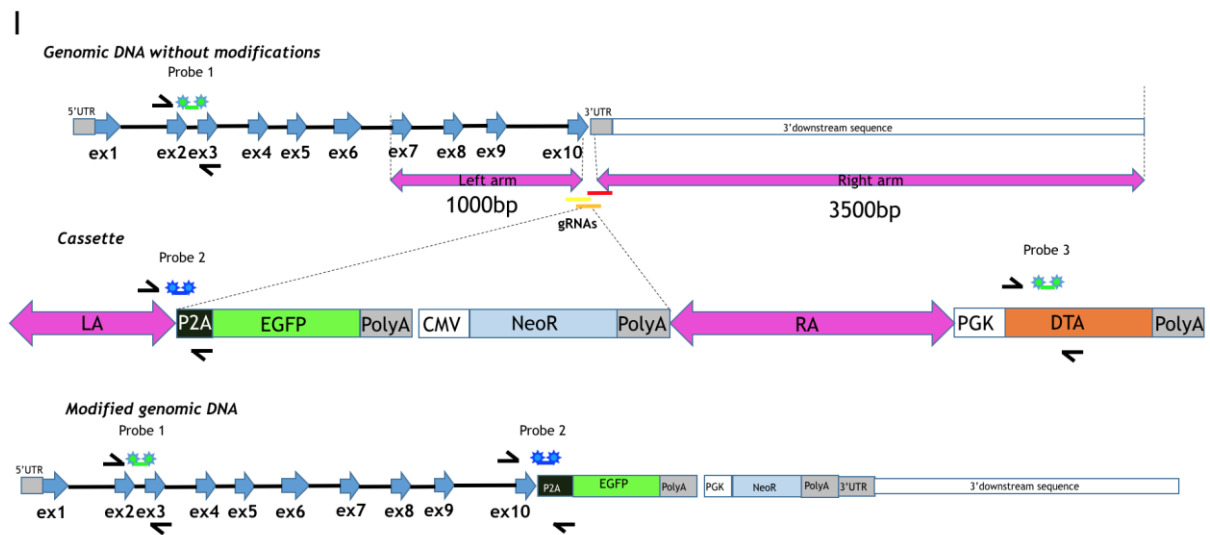

CRISPR/Cas Gene Editing Technology. G3 (Bethesda). 2016 Jun 1;6(6):1787-92.

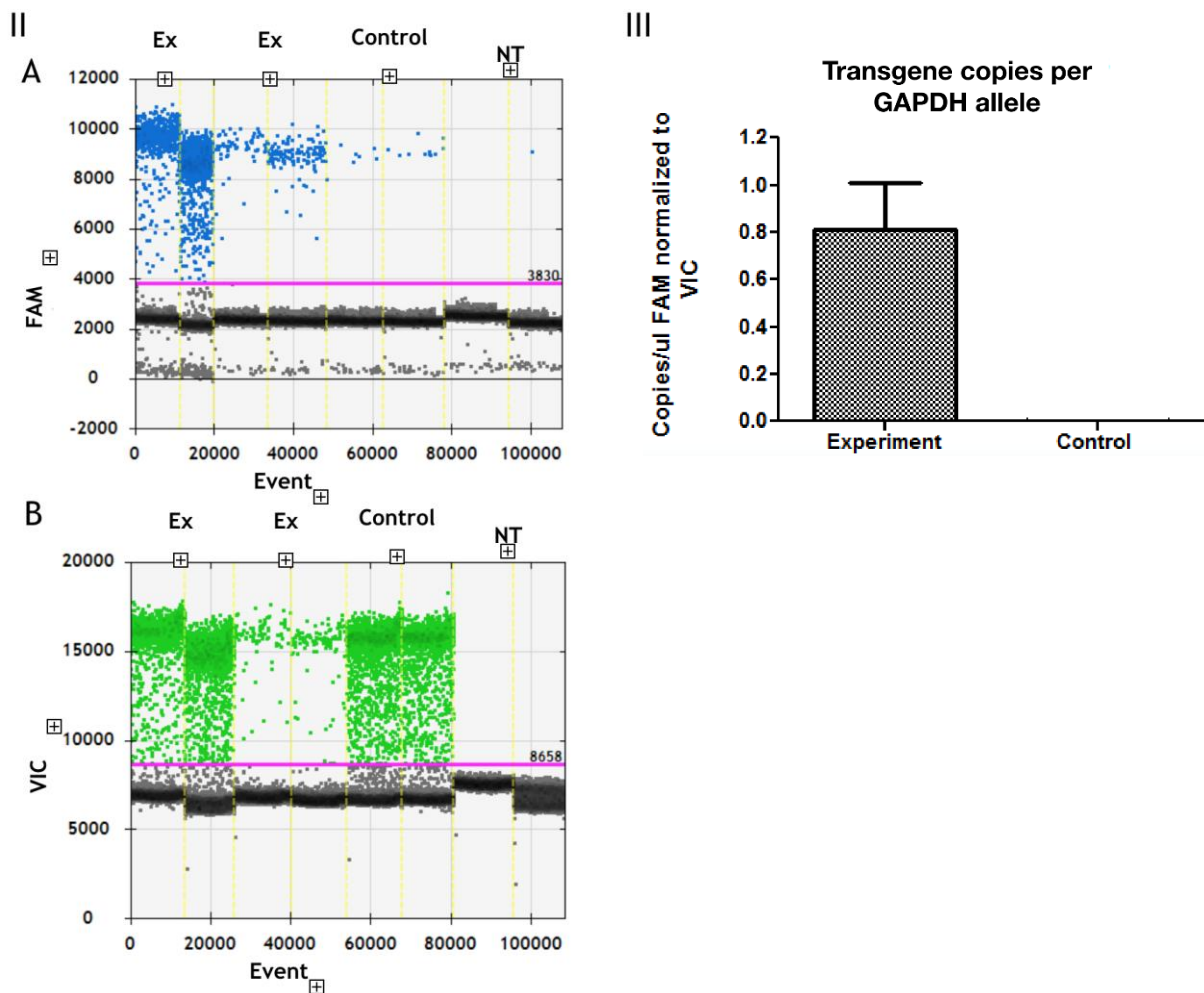

Supplement: Supplementary file 6 [file f1000research-7-16408-s0005.tgz › 3ef9b62d-b78a-457c-a526-6173d0efbccd.pdf]

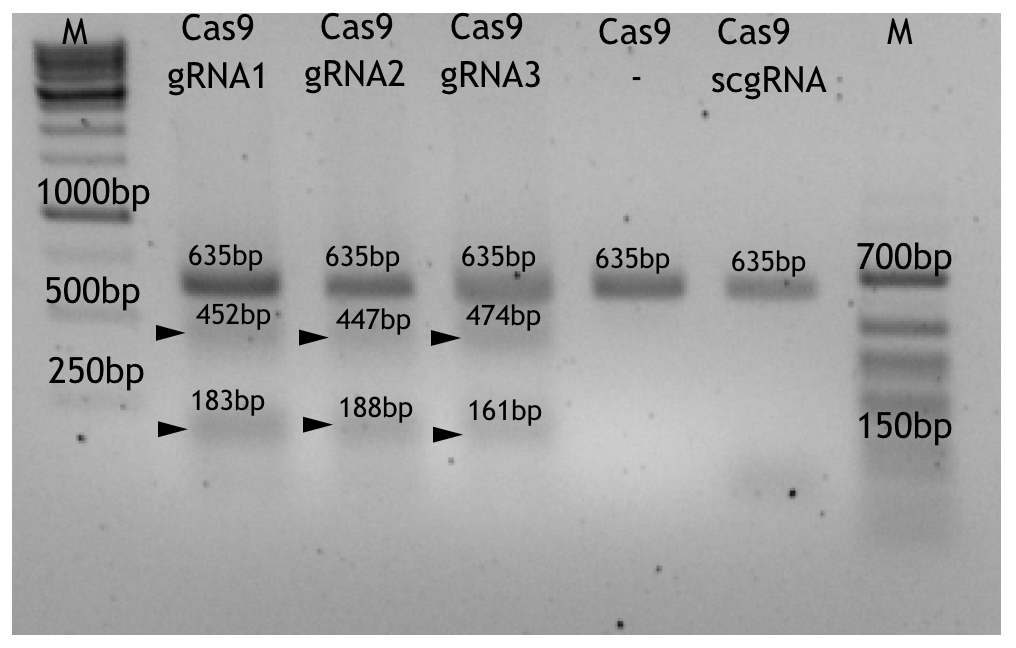

Supplement: Supplementary file 7 [file f1000research-7-16408-s0006.tgz › 30cbc040-7079-439f-930d-ab519a2e538c.png]

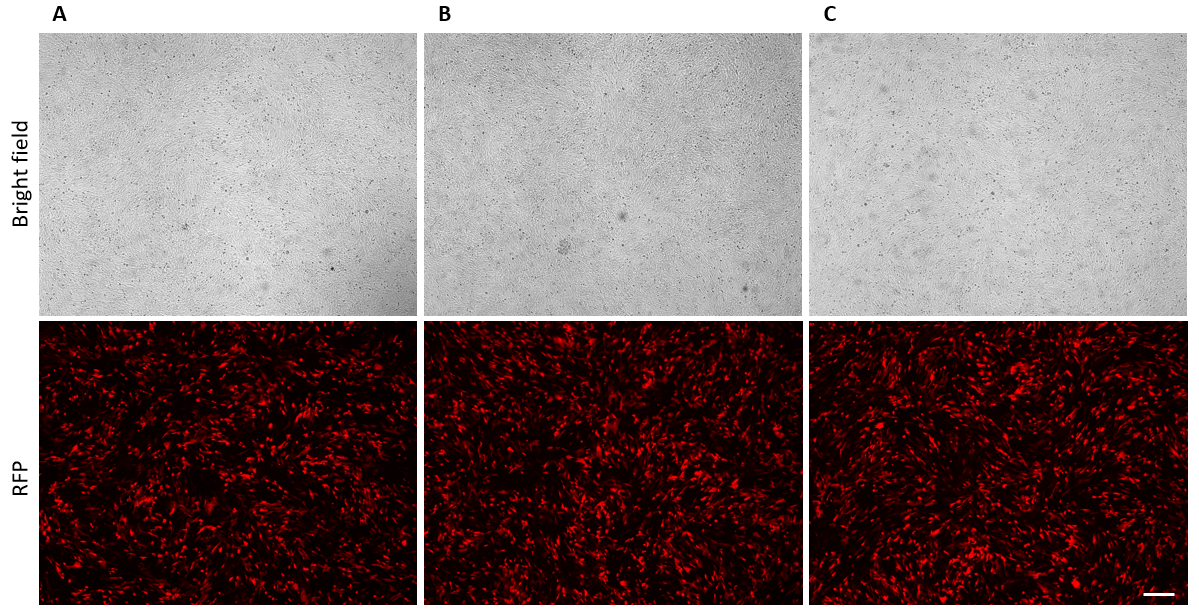

Supplement: Supplementary file 8 [file f1000research-7-16408-s0007.tgz › 52da190e-cb4b-413e-a969-ab6e224ca5f9.png]

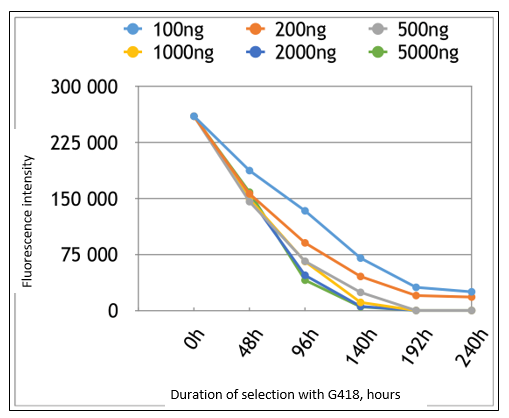

Supplement: Supplementary file 9 [file f1000research-7-16408-s0008.tgz › d2fab492-fc3e-481d-90e1-b732548297b4.png]

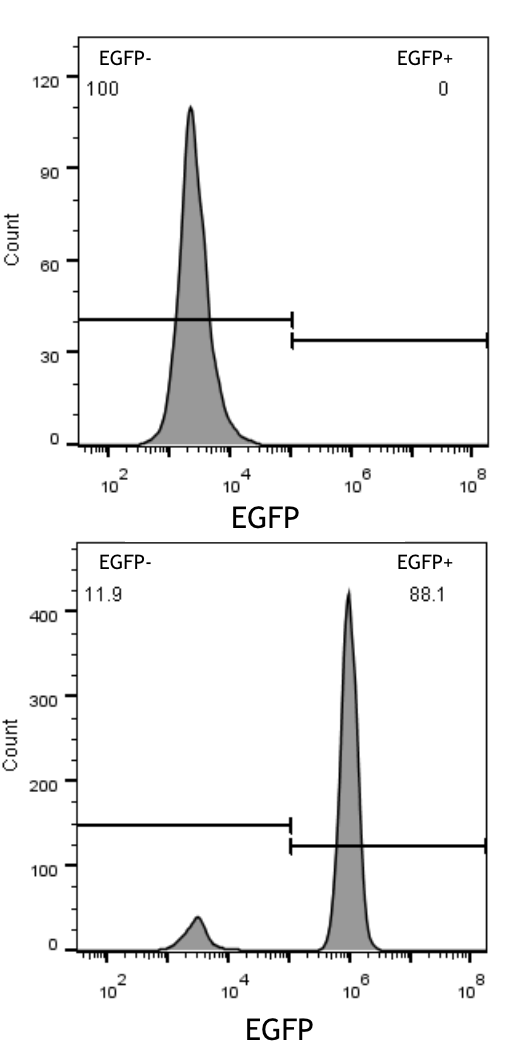

Supplement: Supplementary file 10 [file f1000research-7-16408-s0009.tgz › 0ce9f46d-92af-409e-9fe0-36ab6946ec8b.png]

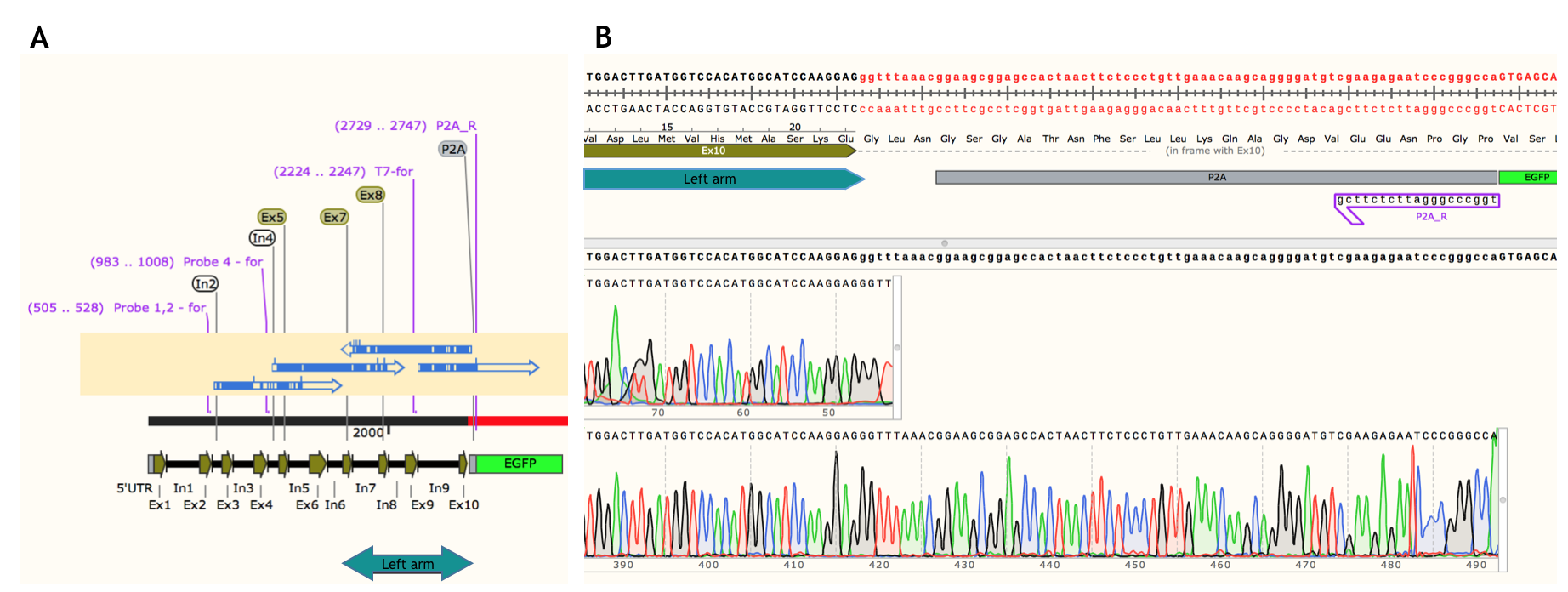

Supplement: Supplementary file 11 [file f1000research-7-16408-s0010.tgz › fc5e91e6-4abc-412c-8aad-0c104a388b55.png]
