# Supplementary material for: Successful CRISPR/Cas9 mediated homologous recombination in a chicken cell line
Source: F1000Res. 2018 May 30;7:238. Originally published 2018 Feb 28. [Version 2] doi: 10.12688/f1000research.13457.2 (PMC6008848; doi:10.12688/f1000research.13457.2)
Supplement: Peer review history

# Peer review: Referee response for version 2

**DOI:** 10.5256/f1000research.16408.r34500

**Published:** 2018-07-02

**Authors:** Lei Qi

**Affiliations:** 1 Departments of Bioengineering and Chemical and Systems Biology, Stanford University Medical Center, Stanford, CA, 94305, USA

I have no further comments to make.

I have read this submission. I believe that I have an appropriate level of expertise to confirm that it is of an acceptable scientific standard.

# Peer review: Referee response for version 2

**DOI:** 10.5256/f1000research.16408.r34805

**Published:** 2018-06-15

**Authors:** Fedor Karginov

**Affiliations:** 1 Department of Molecular, Cell and Systems Biology, University of California, Riverside, Riverside, CA, USA

In this manuscript, the authors present a successful CRISPR/Cas9-HDR workflow in a chicken cell line for expression of eGFP as a bicistronic fusion behind endogenous GAPDH under its promoter. The work is generalizeable to other expression/tagging uses of CRISPR/Cas9 with homologous recombination, and will be of interest to the community working on genetic modification of this organism. The experiments are generally well-conceived and executed, however some concerns are still raised:

 The authors correctly point out that the introduced eGFP is promotorless and is expressed together with GAPDH. However, they also introduced a CMV promoter with the neo marker directly after the GAPDH locus, which creates the possibility of transcriptional interference and/or epigenetic silencing of the locus, since the whole gene appears relatively small, and the endogenous GAPDH promoter is nearby. This, and the modification of the locus in general, prompts the verification of GAPDH expression – do its levels change relative to controls? Does the CMV promoter expose it to silencing after prolonged culture? The authors should discuss modifications to the procedure that would allow for excision of the CMV-neo marker after successful integration (by loxP, for example).

 The incomplete labeling of Fig. S1A,B,C prevents interpretation of the integration events – are these mostly monoallelic or biallelic events? Derivation of clonal colonies may be necessary to fully address this question.

 Many of the figure legends require much more information to interpret:

- Fig. S1A, B, C – what samples are in each of the lanes?
- Fig S6 requires labels and a better legend. What cell populations are on the top and on the bottom –before and after selection?
- Fig. S7 requires a better description/legend. Which components of A) are relevant to the authors statements in the text? What are the sequences shown in B), and which primer sequencing are represented in the chromatographs? Was a single clonal colony raised and selected for sequencing, or is this a PCR product amplified from the bulk selected DNA?

Minor concerns:

- Fig S2 I: what does the reference at the bottom mean?
- Fig. 3 shows microscopy, but the text (Results, “Homologous recombination…”) gives a measurement of GFP+ cells – 0.5% - how was the quantification done? These results should be presented directly.
- Methods, In vitro cleavage by Cas9 – it would be useful to include the concentrations of gRNA, Cas9 and target DNA, as well as the reaction conditions.
- Methods, first paragraph – the “Bai Y” citation is incomplete.
- Methods, “Targeting vector design” – was the DTA sequence inserted or already present in the parental LSLCas9-Rosa26TV plasmid?
- Methods, “PCR analysis”, third paragraph – the authors are likely referring to Fig. S1D for primers pair 3 as well.
- Results, “Targeting 3’ UTR..” – Fig. S2 is likely referenced by mistake.
- Table 2: Primer and probe sequences for ddPCR probes 1 and 2 are listed, but not probe 3 as described in the text.
- Results, “Homologous recombination…” – Fig. S6 does not show PCR analysis results.

I have read this submission. I believe that I have an appropriate level of expertise to confirm that it is of an acceptable scientific standard.

# Peer review: Referee response for version 1

**DOI:** 10.5256/f1000research.14613.r32664

**Published:** 2018-05-02

**Authors:** Lei Qi

**Affiliations:** 1 Departments of Bioengineering and Chemical and Systems Biology, Stanford University Medical Center, Stanford, CA, 94305, USA

The work is a confirmation that the CRISPR/Cas9 system and a template DNA design can be technically used in a chicken cell line for inserting a reporter gene into a genomic locus. The authors demonstrated that insertion of an EGFP gene cassette into the 3’UTR of the endogenous GAPDH gene with an efficiency of 0.5%, and 89% with selection with antibiotic. The authors provided a detailed description of the protocol, which is helpful for other researchers to follow. However, the work remains a proof-of-principle demonstration that the CRISPR+DNA donor can work in a chicken cell line, the efficiency is likely not high enough for applications. The authors didn’t spend efforts to improve the efficiency, nor investigate what might be the causes of the low efficiency. The protocol used in the chicken cell line is largely taken from the one that is used in mammalian cells, including the human codon optimized Cas9, and mammalian promoters for expressing Cas9 and gRNA. To make a convincing case that their method is “robust”, they should test inserting more genes (not only a reporter gene) into more genomic loci (not only GAPDH). Because of this, it remains a question if the described method is robust enough for inserting any gene of interest into any genomic locus of interest. The suggested next-step experiments include optimizing the method to increase the efficiency, testing more genomic loci, testing gene insertion with different lengths, and testing in the chicken primodial germ cells that will be useful for the applications. Other suggestions for improvement:

1. While the authors demonstrate inserting a promoterless gene into the genomic under the control of an endogenous promoter, they also claimed potential tissue-specific gene expression. However, they didn’t prove that their method can result in a cell line that exhibits tissue-specific gene expression.
2. The efficiency of gene cutting in vitro is lower than expected, only 1.8%. What causes this low efficiency? In general, the authors should provide a detailed discussion of the generally low efficiency observed both in vitro and in vivo, which will be the primary targets for improvement. 
3. The authors should mention one additional advantage of their use of the endogenous promoter to express inserted genes, which is less likely be epigenetically silenced. From their data, they observed a relatively long-term (1 month) expression from the endogenous GAPDH promoter. This can be an advantage for long-term stable expression of pharmaceutical proteins compared to using an exogenous promoter that can be epigenetically silenced over time.
4. There is not off-target assay to characterize the real off-target sites from the computational ones. Also, what is NHEJ rate in the correctly EGFP inserted cells?  Are there alleles also show indel editing via NHEJ? It would be useful to understand the NHEJ vs. HDR landscape in a chicken cell line. 
5. The authors should try additional experiments to improve the efficiency. For example, they can try to reduce the number of plasmids used for transfection, test methods such as RNP delivery or using Cas9 mRNA, use a chicken codon-optimized Cas9 and chicken promoters to express Cas9 and gRNA, and re-design the 5’ and 3’ homologous arms. Via these experiments, it may become a more useful resource to understand what might lead to better HDR efficiency in a chicken cell line.

I have read this submission. I believe that I have an appropriate level of expertise to confirm that it is of an acceptable scientific standard, however I have significant reservations, as outlined above.

# Response

**Published:** 2018-05-29

**Authors:** Ekaterina Antonova

**Affiliations:** Moscow Institute of Physics and Technology, Russian Federation

1. Yes, we tell that potentially the method can be used for integration in a tissue-specific locus. The next step will be to apply the approach for integration under a germ cell-specific promoter in PGC cells.

 2. The low efficiency can be explained by several reasons: we were restricted by 50–100 bp sequence of genomic DNA for selection of gRNAs; the area of targeting is the beginning of 3’UTR, which usually has a lower GC content and has a lot of repeated elements; also the transfection efficiency of the DF1 cells with multiple plasmids was less than 50%. The 1,8% effectiveness was pointed for T7E assay but not for in vitro assay.

 The following simple steps could be done for improvement of the locus targeting in the cell line:

 (for increasing CRISPR/Cas9 targeting:)

 -Targeting the last exon and mutation of PAM in HDR cassette instead of targeting the beginning of 3'UTR in the case when there are no appropriate predicted gRNAs. 

 -Increasing of transfection effectiveness (usage of all-in one vector or minicircles, usage of chicken U6 promoter, usage of RNA/RNP delivery for the instruments, etc).

 (for HDR improvement:) 

 -Usage of longer or smaller homology arms. The optimal length of arms could vary in depends on targeting locus.

 -Blocking NHEJ pathway in order to increase HDR events.

 3. The advantage was mentioned in the Version 2, the Discussion section: “Usage of an endogenous promoter to express an inserted gene also could prevent its epigenetic silencing. This is very important for long-term stable expression of pharmaceutical proteins and other applications.”

 4. Yes, however from computational analysis we found that none of the off-target sites were present in a known coding sequence of chicken genome. For instance, gRNA2 targets intragenic locus. Thus experimental off-target estimation is not so actual from point of view of probable harm.  

 Regarding NHEJ and NHEJ vs.HDR:

 We did not estimate NHEJ rate in the correctly EGFP inserted cells, we used ddPCR only for HDR calculation.  We can not analyse the NHEJ vs. HDR landscape like in the publication [1], due to different design of the experiment. In our study long homology arms and long integrated sequences were used that did not allow us to estimate HDR and NHEJ simultaneously.

 Different integrations might occur at two genomic alleles in a single cell. Some EGFP+ cells could have the correct knock-in in at least one allele. Another allele can be unchanged or can contain NHEJ.

 5. There are a lot of parameters for changing in order to improve gene targeting efficiency. Some of them has been already optimized for chicken cells in literature. For instance, U6 promoter yields 4-fold higher gRNA expression than the human U6 in DF1 cell line [2]. We have tested gRNAs delivery in plasmids or amplicons in the cell line, Cas9 with different nuclear localisation signals (SV40 and nucleoplasmin) and several ratios of the instruments [data not shown]. The best conditions were taken to be presented in the article. The aim of the study was not to optimise Cas9 mediated targeting. We wanted to demonstrate that the approach with negative and positive selection works in chicken cells allowing to obtain modified cell line even when targeting effectiveness is low that is expected in the case of PGC cells. We suppose that the results can be improved. For example, variation of different sizes for the 5’ and 3’ homologous arms could increase the quantity of desired cells before enrichment etc. We agree with you that our method can not be called as a robust. It is a possible way for a gene insertion under control of a desired endogene promoter in chicken cells. In further researches the methods should be applied in PGC cells. 

 [1].  Systematic quantification of HDR and NHEJ reveals effects of locus, nuclease, and cell type on genome-editing Yuichiro Miyaoka, Jennifer R. Berman, Samantha B. Cooper, Steven J. Mayerl, Amanda H. Chan, Bin Zhang, George A. Karlin-Neumann & Bruce R. Conklin.

 [2]. Optimization of CRISPR/Cas9 genome editing for loss-of-function in the early chick embryo. ShashankGandhiMichael L.PiacentinoFelipe M.VieceliMarianne E.Bronner

# Peer review: Referee response for version 1

**DOI:** 10.5256/f1000research.14613.r31286

**Published:** 2018-03-12

**Authors:** Zhiying Zhang, Kun Xu

**Affiliations:** 1 Northwest A&F University, Yangling, China

Ekaterina Antonova, ** *et al.* reported their successful integration of " P2A-eGFP-CMV-NeoR " cassette in chicken DF-1cells, achieving eGFP expression under control of the endogenous GAPDH promoter. As the authors claimed, their approach may be used to insert genes of interest under control of tissue-specific promoters for further producing genetically modified birds. The experiments were well designed and the paper was well written.  

 However, there are still some concerns:

 1. Page 3, in the Introduction section, the authors claimed that "Meanwhile CRISPR/Cas9 was used only to knock out genes in poultry". Actually, HDR-based gene editing and integration have also been reported  previously as the authors cited (Wang *et al.*,2017, Dimitrov *et al.,* 2016; page 10) in the Discussion section.

 2. Although the guide RNA (gRNA) is also named single guide RNA (sgRNA), it would be better for the authors to use gRNA or sgRNA consistently through the whole paper.

*3. *Page 7, the authors claimed "FACS analysis showed 90% of eGFP positive cells in the cell population(Figure S6)",  but the data as Figure S6 shown was "EGFP ^+^, 88.1". Nevertheless, page 10, in the Discussion section, the authors again claimed " Using drug selection, we achieved up to 89% targeted integration". In my opinion, they mean the same data. Please check it.

 4. The *in vitro *assay demonstrated all 3 gRNAs functioned (Figure S3), but the T7E1 assay suggested only gRNA2 functioned in cells (Figure2).  Thus, the *in vitro* assay is of no sense and is no longer needed. As a suggestion, the authors may use surrogate reporters ^1^^,^^2^ for future validation of gRNA activity.

 5. Besides, the off-target effect of gRNA2 was not assessed.

 6. As the authors discussed in the Discussion section (page 9), " transfection efficiency of the DF1cells with multiple plasmids was less than 50%". Regarding that the Cas9 gene, the U6-gRNA expression cassette and the RFP marker gene can be easily cloned together within one single plasmid, why did the authors use 3 separate plasmids?

 7. One more concern. The bi-allelic integration in mammalian cells is usually very low. However, here both the PCR and ddPCR analysis implied that all copies (alleles) of GAPDH gene were integrated at the 3'-end with the intent eGFP cassette, after the enrichment.

  Although the authors described that " as it is known that birds have one copy of the gene (GAPDH, page7)", I think it is a pair of alleles but not a single GAPDH gene. Thus, I am wondering that how the mono-allelic integration became bi-allelic after the selection? By HR and the untargeted allelic will gain the integration of eGFP from the targeted one?

We have read this submission. We believe that we have an appropriate level of expertise to confirm that it is of an acceptable scientific standard.

## References

[1] RamakrishnaSChoSWKimSSongMGopalappaRKimJSKimH: Surrogate reporter-based enrichment of cells containing RNA-guided Cas9 nuclease-induced mutations. *Nat Commun*.2014;5: 10.1038/ncomms4378 3378 10.1038/ncomms4378 24569644

[2] XuKRenCLiuZZhangTZhangTLiDWangLYanQGuoLShenJZhangZ: Efficient genome engineering in eukaryotes using Cas9 from Streptococcus thermophilus. *Cell Mol Life Sci*.2015;72(2) : 10.1007/s00018-014-1679-z 383-99 10.1007/s00018-014-1679-z 25038777PMC11113816

# Response

**Published:** 2018-05-26

**Authors:** Ekaterina Antonova

**Affiliations:** Moscow Institute of Physics and Technology, Russian Federation

1. The sentence: "Meanwhile CRISPR/Cas9 was used only to knock out genes in poultry» is about researches that resulted in generation of transgenic chicken (F1 from gremlin chimera) with application of CRISPR/Cas9 technology.  Still there are no data about a genetically modified bird with a gene integration performed with the CRISPR/Cas9. Wang et al.,2017 made research with CRISPR/Cas9-mediated integration in cell culture and Dimitrov et al., 2016 obtained gremline chimeras.

 2. We named all guide RNAs as gRNA through the paper as you recommended.

 3. Yes, it is the same data. We rounded the number consistently in the page 7 and the discussion section.

 4. We will try surrogate reporters in future researches as you advised.

 5. Yes, however we made a bioinformatic analysis and found that none of the off-target sites were present in a known coding sequence of chicken genome. Thus experimental off-target estimation is not so actual for the guides.

 6. Three separate plasmids were used to optimise several conditions that can influence on gene targeting efficiency. We tested different ratios of Cas9 and gRNA. Also we tested amplicons and plasmids for gRNAs delivery. The best conditions were selected for HDR and were presented in the article. RFP was used for cotransfection. We agreed with you that usage one plasmid with all components could increase the number of transfected molecules of instruments per cell and thereby could increase targeting effectiveness. Alternatively mini-circles can be used for the same purpose.

 7. We mentioned about one copy of the gene, because genomes of other animals have different number of GAPDH pseudogenes [Yuen-Jong Liu et al., 2009]. However one copy of GAPDH implies two alleles. PCR analysis does not allow to answer the question bi-allelic or mono-allelic integration happened, the amplicon of the exact size can be amplified in any case of the correct insertion. Based on ddPCR data we calculated the ratio of copies of transgene per one allele of GAPDH gene in total. In the figure S2.III there is a misprint in the title. It should be “Transgene copies per GAPDH allele” instead “Transgene copies per genome”. The title was modified in the version 2. EGFP-positive cells and Geneticin resistant cells could have monoallelic or biallelic integration. The ratio of transgene per genome  is 1,6±0,4 copies based on ddPCR analysis.
